# Supplementary material for: Antibiofilm Effect of Biogenic Silver Nanoparticles Combined with Oregano Derivatives against Carbapenem-Resistant Klebsiella pneumoniae
Source: Antibiotics (Basel). 2023 Apr 14;12(4):756. doi: 10.3390/antibiotics12040756 (PMC10135348; doi:10.3390/antibiotics12040756)
Supplement: Supplementary file 1 [file antibiotics-12-00756-s001.zip › antibiotics-2317943-supplementary.pdf]

Supplementary Material

Antibiofilm effect of biogenic silver nanoparticles combined with oregano derivatives against carbapenem-resistant *Klebsiella pneumoniae*

Sara Scandorieiro, Franciele Maira M. B. Teixeira, Mara C. L. Nogueira, Luciano A. Panagio, Admilton G. de Oliveira, Nelson Durán, Gerson Nakazato, Renata K. T. Kobayashi

Content list of this supplementary file

Figure S1. Size distribution by intensity (%) of bioAgNP provided by photon correlation spectroscopy; average diameter of nanoparticles was 84.10 nm and the polydispersity index (PDI) was 0.269.

Figure S2. Zeta potential distribution of bioAgNP, which average value was -15.9 mV.

Figure S3. Calibration curve used to determine the concentration of silver in bioAgNP after washing steps. Linear calibration curve was obtained of intensities (cps/µA) measured by Energy Dispersive X-ray Fluorescence Spectrometer EDX-7000 in four-point calibration solutions. The silver nitrate concentrations of standard solutions were as follow: 1 mM, 5 mM, 10 mM, and 15 mM. The silver concentration in washed bioAgNP was 6 mM and this value was determined by linear regression analysis.

Table S1. Antibiofilm effect of all tested concentrations of oregano-derivatives and bioAgNP against biofilms growth in microtiter plates, which were evaluated at early stage of biofilm formation.

Table S2. Antibiofilm effect of all tested concentrations of oregano-derivatives and bioAgNP against biofilms growth in microtiter plates, which were evaluated at preformed biofilm condition.

Figure S4. Effect of oregano-derived compounds and bioAgNP, individually and in combination, on biofilm growth of Enteroaggregative Escherichia coli (EAEC 042) and KPC-producing Klebsiella pneumoniae evaluated at early stage of biofilm formation. Biofilm amount is represented in terms of biofilm total biomass and metabolic activity, which were measured using crystal violet-staining and MTT assay respectively, after 24 h-biofilm formation. For each bacterium, oregano-derivatives and bioAgNP were tested at same concentrations individually and in combinations. Control indicates bacterial biofilm growth with no antimicrobial. (A) EAEC 042 exposed to OEO at 0.15 mg/mL, Car at 0.15 mg/mL, Thy at 0.12 mg/mL, and bioAgNP at 0.25 µg/mL, individually or in binary combinations. (B) KPC-producing K. pneumoniae exposed to OEO at 0.3 mg/mL, Car at 0.15 mg/mL, Thy at 0.12 mg/mL, and bioAgNP at 0.98 µgmL, individually or in binary combinations. Values

of biomass and viability (%) are the mean ± standard deviation. * Indicates a statistically significant difference ($p<0.05$, Kruskal-Wallis test) between treatment and untreated control in terms of biomass or metabolic activity.

Figure S5. Effect of oregano-derived antibacterials and bioAgNP, individually and in combination, on biofilm growth of Enteroaggregative Escherichia coli (EAEC 042) and KPC-producing Klebsiella pneumoniae evaluated at later stage under preformed biofilm condition. Biofilm amount is represented in terms of metabolic activity, which were measured using MTT assay after 24 h of treatment of 24 h-preformed biofilm; biofilm viability was measured after total 48 h-biofilm formation. For each bacterium, oregano-derivatives and bioAgNP were tested at same concentrations individually and in combinations. Control indicates bacterial biofilm growth with no antimicrobial. Red bars indicate EAEC 042 exposed to OEO at 0.30 mg/mL, Car at 0.31 mg/mL, Thy at 0.25 mg/mL, and bioAgNP at 7.88 µg/mL, individually or in binary combinations. Gray bars indicate KPC-producing K. pneumoniae exposed to OEO at 0.30 mg/mL, Car at 0.15 mg/mL, Thy at 0.06 mg/mL, and bioAgNP at 1.97 µg/mL, individually or in binary combinations. Values of viability (%) are the mean ± standard deviation. * Indicates a statistically significant difference ($p<0.05$, Kruskal- Wallis test) between treatment and untreated control in terms of metabolic activity.

Table S3 – Quantitative reduction of violacein produced by Chromobacterium violaceum treated with oregano-derived antibacterials and bioAgNP, alone and in combination. Viable cells number (log CFU/mL) of untreated (control) and treated-C. violaceum are also shown.

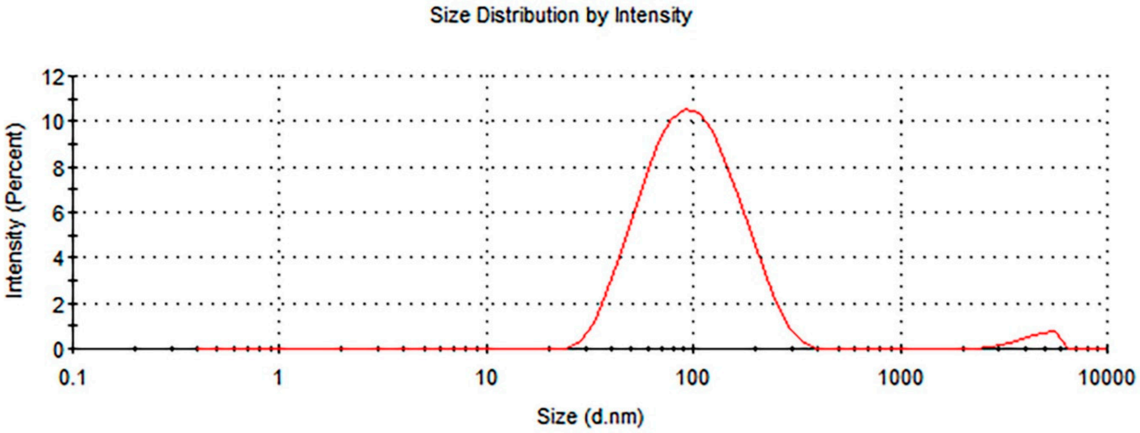

Figure S1. Size distribution by intensity (%) of bioAgNP provided by photon correlation spectroscopy; average diameter of nanoparticles was 84.10 nm and the polydispersity index (PDI) was 0.269.

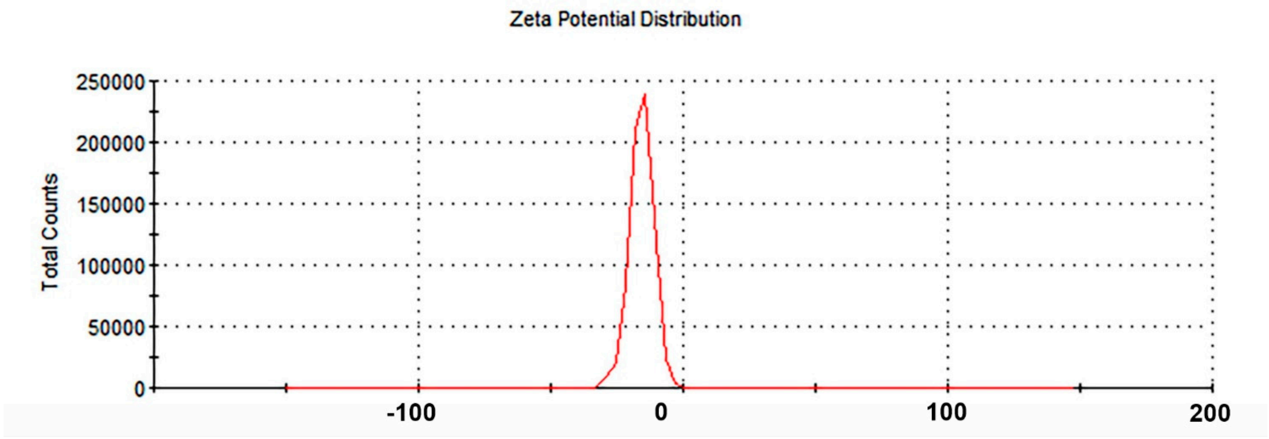

Figure S2. Zeta potential distribution of bioAgNP, which average value was -15.9 mV.

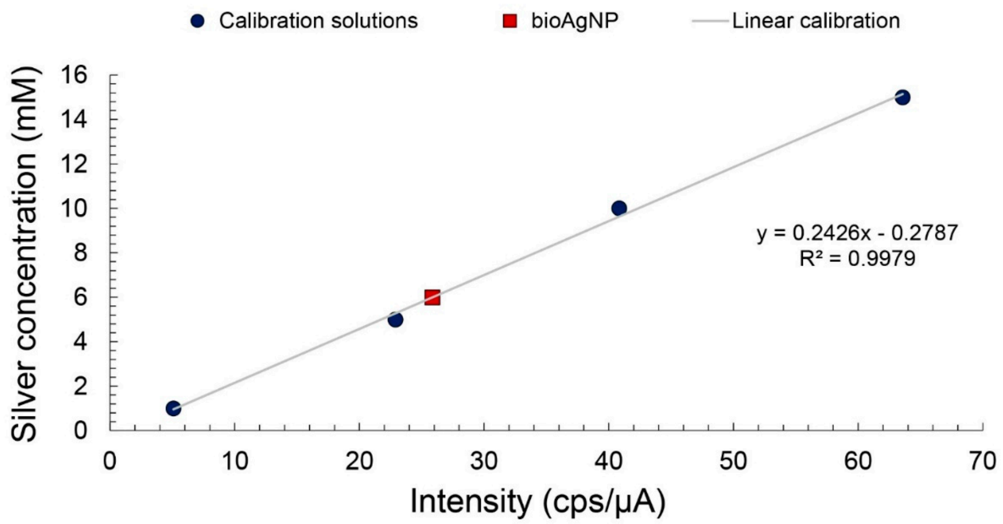

Figure S3. Calibration curve used to determine the concentration of silver in bioAgNP after washing steps. Linear calibration curve was obtained of intensities (cps/µA) measured by Energy Dispersive X-ray Fluorescence Spectrometer EDX-7000 in four-point calibration solutions. The silver nitrate concentrations of standard solutions were as follow: 1 mM, 5 mM, 10 mM, and 15 mM. The silver concentration in washed bioAgNP was 6 mM and this value was determined by linear regression analysis.

Table S1. Antibiofilm effect of all tested concentrations of oregano-derivatives and bioAgNP individually against Enteroaggregative *Escherichia coli* (EAEC 042) and *Klebsiella pneumoniae* (KPC) biofilms growth in microtiter plates, which were evaluated under inhibition condition.

	EAEC		KPC			EAEC		KPC			EAEC		KPC			EAEC		KPC	
**OEO mg/ml**	**CV**	**MTT**	**CV**	**MTT**	**Car mg/ml**	**CV**	**MTT**	**CV**	**MTT**	**Thy mg/ml**	**CV**	**MTT**	**CV**	**MTT**	**bio AgNP µg/mL**	**CV**	**MTT**	**CV**	**MTT**
**9.5**	-	-	-	-	**9.76**	-	-	-	-	**2**	-	-	-	-	**7.88**	NT	NT	-	-
**4.75**	-	-	-	-	**4.88**	-	-	-	-	**1**	-	-	-	-	**3.94**	NT	NT	-	-
**2.38**	-	-	-	-	**2.44**	-	-	-	-	**0.5**	-	-	-	-	**1.97**	-	-	-	-
**1.19**	-	-	-	-	**1.22**	-	-	-	-	**0.25**	-	-	-	-	**0.98**	-	-	+	+
**0.59**	-	-	-	-	**0.61**	-	-	-	-	**0.12**	+	+	+	+	**0.49**	+	+	+	+
**0.30**	-	-	+	+	**0.31**	-	-	-	-	**0.06**	+	+	+	+	**0.25**	+	+	+	+
**0.15**	+	+	+	+	**0.15**	+	+	+	+	**0.03**	+	+	+	+	**0.12**	+	+	+	+
**0.07**	+	+	+	+	**0.08**	+	+	+	+	**0.01**	+	+	+	+	**0.06**	+	+	+	+
															**0.03**	+	+	NT	NT
															**0.01**	+	+	NT	NT

MTT, dimethylthiazol diphenyl tetrazolium bromide assay to detect biofilm metabolic activity. CV, crystal violet-staining performed to measure total biofilm biomass.
OEO, Oregano essential oil. Car, Carvacrol. Thy, Thymol. bio-AgNP, biogenic silver nanoparticles.
- Indicates “no biofilm metabolic activity” and “at least 90% (of reduction in total biofilm biomass” detected after 24h of treatments; for each compound, comparative analysis among different concentrations of treatment and untreated control showed statistically significant difference in terms of biomass or metabolic activity of biofilm ($p<0.05$, Kruskal–Wallis test).
+ Indicates “active biofilm metabolic activity” and “biofilm biomass production”; there is no difference between treated samples and untreated control in terms of biomass or metabolic activity of biofilm.
NT indicates “concentration not tested”.

Table S2. Antibiofilm effect of all tested concentrations of oregano-derivatives and bioAgNP individually against Enteroaggregative *Escherichia coli* (EAEC 042) and *Klebsiella pneumoniae* (KPC) biofilms growth in microtiter plates, which were evaluated at preformed biofilm condition.

	MTT			MTT			MTT		bio	MTT	
**OEO mg/ml**	**EAEC**	**KPC**	**Car mg/ml**	**EAEC**	**KPC**	**Thy mg/ml**	**EAEC**	**KPC**	**AgNP µg/mL**	**EAEC**	**KPC**
**9.5**	-	-	**9.76**	-	-	**2**	-	-	**126**	-	-
**4.75**	-	-	**4.88**	-	-	**1**	-	-	**63**	-	-
**2.38**	-	-	**2.44**	-	-	**0.5**	-	-	**31.5**	-	-
**1.19**	-	-	**1.22**	-	-	**0.25**	+	-	**15.75**	+	-
**0.59**	-	-	**0.61**	-	-	**0.12**	+	+	**7.88**	+	-
**0.30**	+	+	**0.31**	+	-	**0.06**	+	+	**3.94**	+	+
**0.15**	+	+	**0.15**	+	+	**0.03**	+	+	**1.97**	+	+
**0.07**	+	+	**0.08**	+	+	**0.01**	+	+	**0.98**	+	+

MTT, dimethylthiazol diphenyl tetrazolium bromide assay to detect biofilm metabolic activity.

OEO, Oregano essential oil. Car, Carvacrol. Thy, Thymol. bio-AgNP, biogenic silver nanoparticles.

- Indicates "at least 95% ($p<0.05$) of reduction in metabolic activity of bacterial preformed biofilm" after 24h of treatments; for each compound, comparative analysis among different concentrations of treatment and untreated control showed statistically significant difference in terms of metabolic activity of biofilm ($p<0.05$, Kruskal-Wallis test).

+ Indicates "active biofilm metabolic activity"; there is no difference between treated samples and untreated control in terms of biomass or metabolic activity of biofilm.

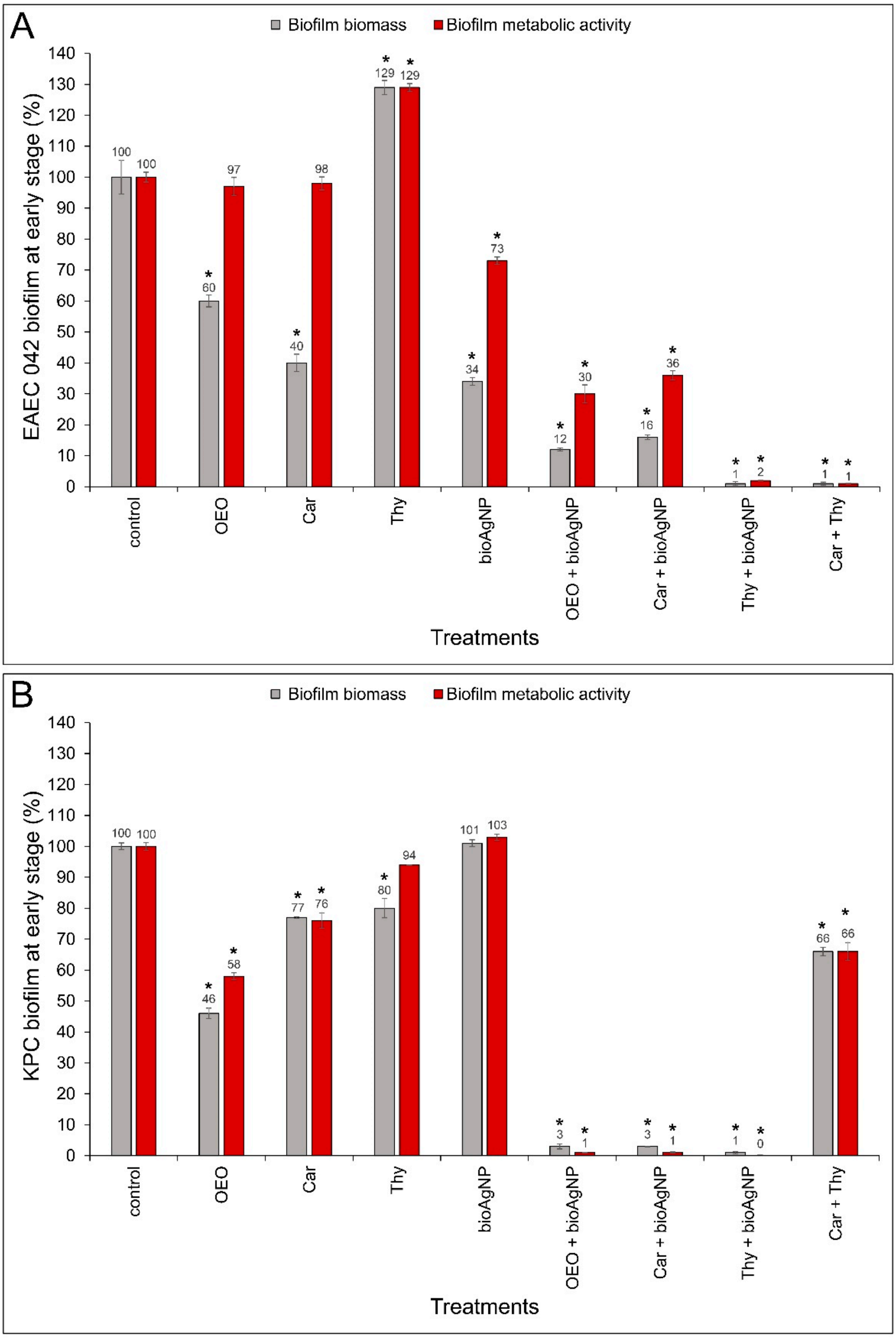

Figure S4. Effect of oregano-derived compounds and bioAgNP, individually and in combination, on biofilm growth of Enteroaggregative Escherichia coli (EAEC 042) and KPC-producing Klebsiella pneumoniae evaluated at early stage of

biofilm formation. Biofilm amount is represented in terms of biofilm total biomass and metabolic activity, which were measured using crystal violet-staining and MTT assay respectively, after 24 h-biofilm formation. For each bacterium, oregano-derivatives and bioAgNP were tested at same concentrations individually and in combinations. Control indicates bacterial biofilm growth with no antimicrobial. (A) EAEC 042 exposed to OEO at 0.15 mg/mL, Car at 0.15 mg/mL, Thy at 0.12 mg/mL, and bioAgNP at 0.25 µg/mL, individually or in binary combinations. (B) KPC-producing K. pneumoniae exposed to OEO at 0.3 mg/mL, Car at 0.15 mg/mL, Thy at 0.12 mg/mL, and bioAgNP at 0.98 µgmL, individually or in binary combinations. Values of biomass and viability (%) are the mean ± standard deviation. * Indicates a statistically significant difference ($p<0.05$, Kruskal-Wallis test) between treatment and untreated control in terms of biomass or metabolic activity.

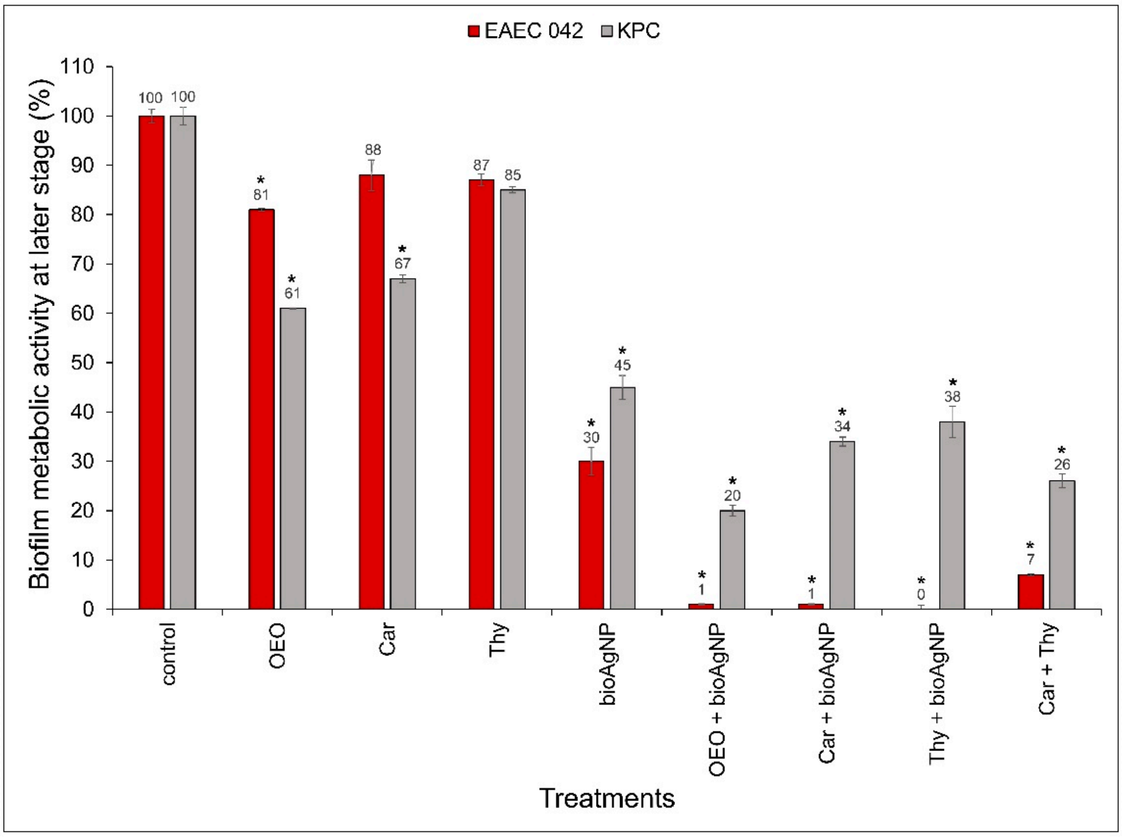

Figure S5. Effect of oregano-derived antibacterials and bioAgNP, individually and in combination, on biofilm growth of Enteroaggregative Escherichia coli (EAEC 042) and KPC-producing Klebsiella pneumoniae evaluated at later stage under preformed biofilm condition. Biofilm amount is represented in terms of metabolic activity, which were measured using MTT assay after 24 h of treatment of 24 h-preformed biofilm; biofilm viability was measured after total 48 h-biofilm formation. For each bacterium, oregano-derivatives and bioAgNP were tested at same concentrations individually and in combinations. Control indicates bacterial biofilm growth with no antimicrobial. Red bars indicate EAEC 042 exposed to OEO at 0.30 mg/mL, Car at 0.31 mg/mL, Thy at 0.25 mg/mL, and bioAgNP at 7.88 µg/mL, individually or in binary combinations. Gray bars indicate KPC-producing K. pneumoniae exposed to OEO at 0.30 mg/mL, Car at 0.15 mg/mL, Thy at 0.06 mg/mL, and bioAgNP at 1.97 µg/mL, individually or in binary combinations. Values of viability (%) are the mean ± standard deviation. * Indicates a statistically significant difference ($p<0.05$, Kruskal- Wallis test) between treatment and untreated control in terms of metabolic activity.

Table S3 – Quantitative reduction of violacein produced by Chromobacterium violaceum treated with oregano-derived antibacterials and bioAgNP, alone and in combination. Viable cells number (log CFU/mL) of untreated (control) and treated-C. violaceum are also shown.

**Antibacterial-treated bacterial sample**	**Antibacterial concentrations**	**Violacein reduction (%)**	**Viable cells (log CFU/mL)**
OEO	0.07 mg/mL	93 ± 0.2	9.4 ± 0.1
Car	0.04 mg/mL	94 ± 0.2	8.9 ± 0.1
Thy	0.06 mg/mL	92 ± 0.3	9.2 ± 0.2
bioAgNP	3.94 µg/mL	81 ± 1.9	9.4 ± 0.1
Thy + bioAgNP	0.008 mg/mL + 0.49 µg/mL	95 ± 0.2	9.2 ± 0.1
Control	no antimicrobial	-	9.1 ± 0.1

Oregano derivatives: OEO (oregano essential oil), Car (carvacrol) and Thy (thymol).
Violacein reduction is expressed in percentage and compared to untreated control. Non-treated control is defined as 100% of violacein production.
± (standard deviation). CFU: colony-forming units.
